# Temperature-Dependent Effects of Eicosapentaenoic Acid (EPA) on Browning of Subcutaneous Adipose Tissue in UCP1 Knockout Male Mice

**DOI:** 10.3390/ijms24108708

**Published:** 2023-05-13

**Authors:** Yujiao Zu, Mandana Pahlavani, Latha Ramalingam, Shasika Jayarathne, Jose Andrade, Shane Scoggin, William T. Festuccia, Nishan S. Kalupahana, Naima Moustaid-Moussa

**Affiliations:** 1 Department of Nutritional Sciences, Texas Tech University, Lubbock, TX 79409, USA; yujiao.zu@ttu.edu (Y.Z.);; 2 Obesity Research Institute, Texas Tech University, Lubbock, TX 79409, USA; 3 Institute of Biomedical Sciences, University of Sao Paulo, Sao Paulo 05508-000, Brazil; 4 Department of Physiology, Faculty of Medicine, University of Peradeniya, Peradeniya 20400, Sri Lanka

**Keywords:** eicosapentaenoic acid, obesity, uncoupling protein 1, beige adipocytes, environmental temperature

## Abstract

Uncoupling protein 1 (UCP1) plays a central role in thermogenic tissues by uncoupling cellular respiration to dissipate energy. Beige adipocytes, an inducible form of thermogenic cells in subcutaneous adipose tissue (SAT), have become a major focus in obesity research. We have previously shown that eicosapentaenoic acid (EPA) ameliorated high-fat diet (HFD)-induced obesity by activating brown fat in C57BL/6J (B6) mice at thermoneutrality (30 °C), independently of UCP1. Here, we investigated whether ambient temperature (22 °C) impacts EPA effects on SAT browning in wild-type (WT) and UCP1 knockout (KO) male mice and dissected underlying mechanisms using a cell model. We observed resistance to diet-induced obesity in UCP1 KO mice fed HFD at ambient temperature, with significantly higher expression of UCP1-independent thermogenic markers, compared to WT mice. These markers included the fibroblast growth factor 21 (FGF21) and sarco/endoplasmic reticulum Ca^2+^-ATPase 2b (SERCA2b), suggesting the indispensable role of temperature in beige fat reprogramming. Surprisingly, although EPA induced thermogenic effects in SAT-derived adipocytes harvested from both KO and WT mice, EPA only increased thermogenic gene and protein expression in the SAT of UCP1 KO mice housed at ambient temperature. Collectively, our findings indicate that the thermogenic effects of EPA, which are independent of UCP1, occur in a temperature-dependent manner.

## 1. Introduction

Obesity is a chronic complex disease, which occurs when energy consumption exceeds expenditure chronically and which is associated with several comorbidities like type II diabetes (T2D) [1], cardiovascular diseases [2], and certain cancers [3]. White adipose tissue (WAT) stores excess energy in the form of triglycerides and secretes several hormones but upon the onset of obesity develops local chronic inflammation. In contrast, brown adipose tissue (BAT) drives adaptive non-shivering thermogenesis in response to cold temperatures and impacts body weight [4]. In rodent models, activation of BAT thermogenesis is involved in reducing diet-induced weight gain [5], and the ablation of BAT is associated with the development of obesity [6]. However, although the presence of BAT in human adults is well-accepted [7], the contribution of BAT to the regulation of body weight and metabolic health is still a matter of debate among researchers [8]. The classical non-shivering thermogenesis occurs not only in BAT but also in beige adipose tissue [9]. Under certain pharmacological and dietary conditions or external stimuli, subcutaneous adipose tissue (SAT) can undergo browning to develop thermogenic brown-like properties, like uncoupling protein 1 (UCP1) expression [10]. Intriguingly, genetic deficiency of BAT in mice increases sympathetic activity to SAT to promote the compensatory recruitment of beige adipocytes [11].

When properly activated, UCP1 catalyzes the leak of protons generated by the electron transport chain to heat production [12]. Independently of UCP1, other thermogenic mechanisms such as the creatine-driven substrate futile cycling [13], the ATP-dependent calcium cycling [14], and endogenous uncoupler N-acyl amino acids [15] have been shown to occur in brown and beige adipocytes. In our current study, to investigate the physiological role of UCP1 and the therapeutic potential of SAT browning in protecting against weight gain, UCP1-knockout (KO) B6 male mice were exposed to either thermoneutral (28–30 °C) or ambient (22 °C) environments. Accumulating evidence has shown that housing UCP1 KO mice at thermoneutrality markedly reduces BAT and SAT thermogenesis and predisposes them to diet-induced obesity (DIO) [16]. Paradoxically, ambient temperature serves as a cold challenge for UCP1 KO mice resulting in increased energy expenditure and resistance to DIO when compared to wild-type (WT) mice [17]. Moreover, the UCP1-independent thermogenesis may have stronger effects in preventing DIO than the classical UCP1-predominant thermogenesis when housing mice at ambient temperature. However, neither the molecular mechanisms nor the regulators have been clearly characterized yet, and identifying them could potentially reveal novel therapeutic targets in treating individuals with obesity.

Dietary long-chain omega-3 polyunsaturated fatty acids (PUFA) such as eicosapentaenoic acid (EPA; 20:5; n-3), the main component in fish oil, is an anti-inflammatory bioactive compound with potential to induce white fat cell browning [18]. We have previously reported that EPA significantly upregulated the mRNA expression levels of key markers of thermogenesis, such as peroxisome proliferator-activated receptor gamma coactivator-1 alpha (PGC1α) and PR domain containing 16 (PRDM16) in HIB 1B clonal brown adipocytes and in the BAT of mice housed at ambient temperature [5]. In addition, utilizing DIO UCP1 KO male mice housed at thermoneutrality, we found that EPA reduced body weight and adiposity and increased BAT PGC1α protein and gene expression, independently of UCP1 [19,20]. Based on the above insights, in the current study, we hypothesized that supplementation with EPA promotes beige adipocyte formation in SAT independently of UCP1 at both ambient and thermoneutral conditions.

To gain mechanistic insights of the EPA effects on SAT thermogenesis and how UCP1 controls obesity resistance temperature-dependently, in the current study, we used WT and UCP1 KO male mice housed at either ambient or thermoneutral environments and supplemented with high-fat diets (HFD) with or without EPA-enriched fish oil. To dissect the role of EPA in promoting beige adipocytes thermogenesis, we further harvested primary SAT adipocytes from WT and UCP1 KO mice treated with or without EPA during differentiation.

We observed the paradoxical obesity resistance of HFD-fed UCP1 KO mice at ambient temperature. Although no significant impact of EPA on body weight and adiposity was observed, insulin resistance and inflammation were attenuated by EPA in both WT and UCP1 KO mice at both temperatures. Importantly, the expression level of genes involved in thermogenesis and lipid metabolism was significantly upregulated at ambient temperature, independently of UCP1. We also found that although EPA enhanced thermogenic gene expression and the respiration capacity of differentiated SAT-derived adipocytes, EPA only upregulated gene and protein expression levels of thermogenic genes at ambient temperature, UCP1 independently. Based on the outcomes of the current study, we demonstrated evidence for the potential use of EPA in combating obesity and improving overall metabolic health via alternative UCP1-independent thermogenesis. 

## 2. Results

### 2.1. UCP1 KO Mice Are Protected from DIO at Ambient Temperature

**Figure 1:**
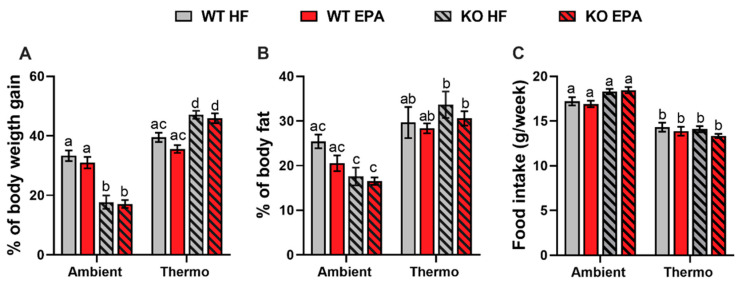
Body weight and fat percentage, and food intake in WT and UCP1 KO mice fed an HFD or EPA-supplemented diet at ambient and thermoneutral (Thermo) conditions. (**A**) Percentage of body weight gain, (**B**) percentage of body fat and (**C**) food intake. Data are expressed as mean ± standard error of mean (SEM); groups represented with different letter indicate significant difference reported by three-way analysis of variance (ANOVA), *p* < 0.05, n = 10–12.

As the most sensitive hallmark regarding energy balance changes, the body weight of mice housed at ambient temperature was lower than the mice at thermoneutrality. Further, the resistance of DIO in UCP1 KO mice was a reproducible phenomenon [21] at ambient temperature. Conversely, after 14 weeks housed at thermoneutrality, UCP1 KO mice had significantly higher weight gain than WT mice (*p* < 0.05) (Figure 1A), suggesting the accumulation of excess energy in fat. Regarding dietary intervention, limited benefits of EPA in reducing obesity in both WT and UCP1 KO mice occurred at thermoneutrality [19]. Similarly, in response to EPA, both WT and UCP1 KO mice gained 4–10% less body weight (Figure 1A) and 6–20% less body fat (Figure 1B) compared to HFD-fed mice at both temperatures, but no significant difference was found. Although food intake did not reveal any differences, mice housed at ambient conditions consumed about 25% more food than those at thermoneutrality (*p* < 0.05), suggesting the compensation for heat loss (Figure 1C). Finally, a significant interaction was observed between temperature and genotype for body weight gain, body fat percentage, and food intake (*p* < 0.01, Table 1).

### 2.2. Effects of EPA and UCP1 Deficiency on Insulin Sensitivity

**Figure 2:**
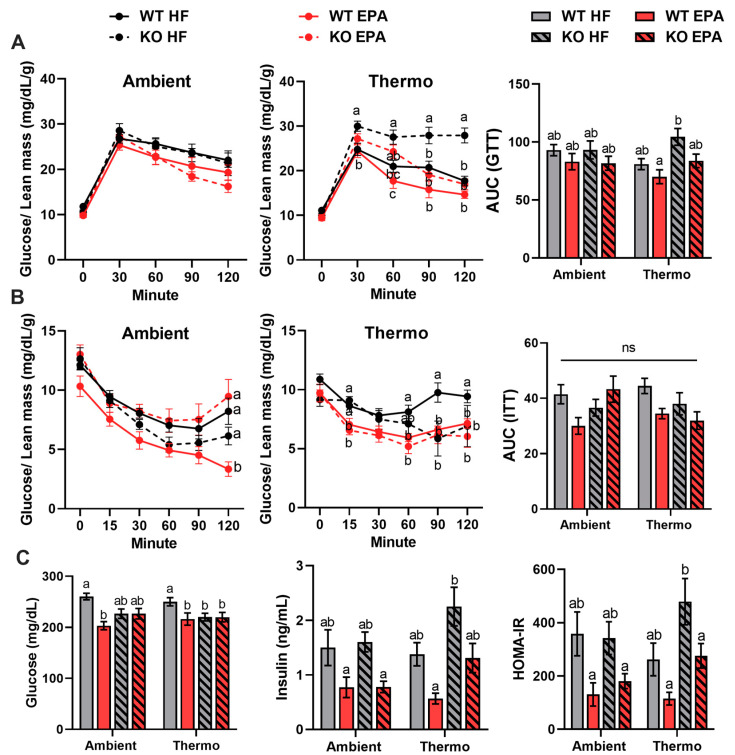
Effects of UCP1 deficiency and temperature on glucose homeostasis and insulin sensitivity in WT and UCP1 KO mice fed an HFD or EPA-supplemented diet. (**A**) GTT and area under the curve (AUC) of GTT, (**B**) ITT, and AUC of ITT and (**C**) basal fasting serum glucose and insulin levels and HOMA-IR of WT and UCP1 KO mice at ambient and thermoneutral conditions. GTT and ITT were normalized to the lean mass of the mice. Data are expressed as mean ± SEM; groups represented with different letter indicate significant difference reported by three-way ANOVA, *p* < 0.05, n = 10. Figures with no letters indicate no statistical (ns) differences across the groups.

We performed glucose and insulin tolerance tests (GTT and ITT, respectively), measured fasting serum insulin levels, and calculated homeostatic model assessment for insulin resistance (HOMA-IR) in WT and KO mice housed at both temperatures to examine the effects of UCP1 deficiency and EPA on glucose metabolism. To compare groups that differ in fat mass, GTT and ITT normalized to the lean mass of mice have been used. Glucose intolerance was not different between genotypes at ambient temperature; however, in association with the increased body weight, it was higher in the UCP1 KO mice compared to the WT mice at thermoneutrality. Importantly, EPA-fed mice exhibited improved glucose clearance compared to the HFD-fed mice in both genotypes and temperatures (Figure 2A). Additionally, there were no differences in ITT between the genotypes in both temperatures (Figure 2B), but EPA significantly increased the insulin sensitivity of mice in both genotypes and temperatures, as indicated by lower basal serum insulin levels and HOMA-IR compared to the HFD-fed mice (Figure 2C). Multifactorial ANOVA revealed a significant main effect for diet (*p* < 0.05) on glucose tolerance and insulin sensitivity, confirming the beneficial effect of EPA on glucose homeostasis (Table 1).

### 2.3. Effects of EPA and UCP1 Deficiency on Metabolic Hormones

**Figure 3:**
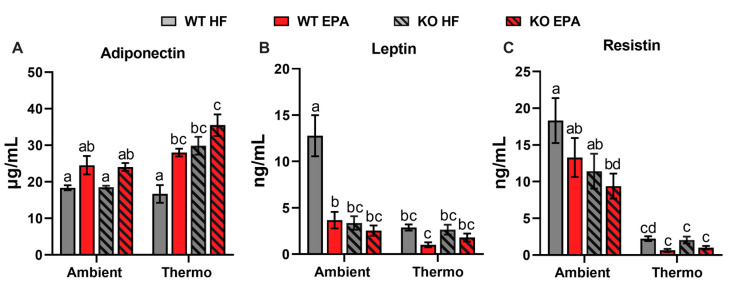
Effects of UCP1 and temperature on serum hormones in WT and UCP1 KO mice fed an HFD or EPA-supplemented diet. (**A**) Serum levels of adiponectin, (**B**) leptin, and (**C**) resistin of mice. Data are expressed as mean ± SEM; groups represented with different letter indicate significant difference reported by three-way-ANOVA, *p* < 0.05, n = 8.

To understand the role of EPA supplementation in adipokine production, serum levels of cytokines involved in inflammation, including adiponectin, leptin, and resistin, were measured. In the HFD groups, there were no differences in serum adiponectin levels between WT and UCP1 KO mice at ambient temperature, whereas, at thermoneutrality, KO mice have two-fold higher adiponectin level than WT mice (*p* < 0.001). Compared to HFD, adiponectin levels were increased in response to EPA in WT and UCP1 KO mice by 34% and 30% at ambient temperature and by 68% (*p* = 0.0049) and 18% at thermoneutrality (Figure 3A). HFD-fed WT mice had significantly higher serum leptin levels compared to all other groups. Corresponding with body fat, the absence of UCP1 caused a significant decrease in leptin levels at ambient temperature but not at thermoneutrality. Additionally, compared to HFD, EPA reduced serum leptin levels in WT and KO mice by 71% (*p* < 0.0001) and 24% at ambient temperature and by 64% and 31% at thermoneutrality, respectively (Figure 3B). Finally, serum resistin levels were decreased in mice housed at thermoneutrality (*p* < 0.001). Additionally, EPA decreased the level of resistin in WT and KO mice by 28% and 18% at ambient temperature and by 73% and 50% at thermoneutrality (Figure 3C). A significant effect of temperature (*p* < 0.0001) and genotype (*p* < 0.05) and the interaction between temperature and genotype (*p* < 0.05) were observed for serum levels of adiponectin, leptin, and resistin (Table 1).

### 2.4. Effects of EPA and UCP1 Deficiency on SAT Browning Temperature-Dependently

**Figure 4:**
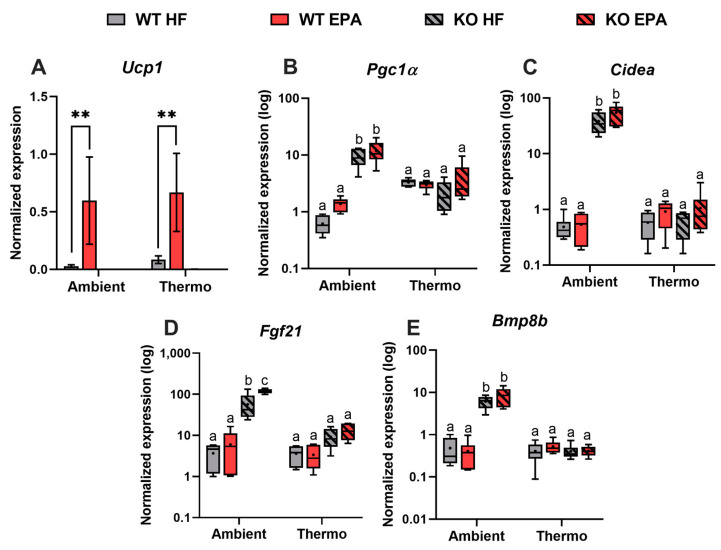
Effects of UCP1 and temperature on thermogenic gene expression levels in the SAT of WT and UCP1 KO mice fed an HFD or EPA-supplemented diet. The gene expression levels for (**A**) *Ucp1*, (**B**) *Pgc1α*, (**C**) *Cidea*, (**D**) *Fgf21*, and (**E**) *Bmp8b*. For (**A**), data were analyzed by *t*-test ** *p* < 0.01. For (**B**–**E**), box plots show the distribution of log2 gene expression level. Data were expressed as mean ± SEM and analyzed by three-way ANOVA with different letters indicating significant difference, *p* < 0.05, n = 6.

We previously demonstrated that EPA increased UCP1 and thermogenic genes in the brown fat of mice maintained at ambient [5] or thermoneutral environments [19]. In the current study, we evaluated the effects of EPA supplementation and UCP1 deficiency on SAT browning, thermogenesis, and lipid metabolism. As expected, compared to an HFD, EPA significantly enhanced Ucp1 mRNA expression in WT mice by 21.1-fold at ambient temperature (*p* < 0.001) and 7.9-fold at thermoneutrality (*p* < 0.001). *Ucp1* mRNA expression levels were undetectable in the UCP1 KO mice (Figure 4A). In addition, based on the regulatory network of thermogenic transcription factors, compared to WT, SAT browning in UCP1 KO mice housed at ambient temperature appeared to be predominantly regulated by *Pgc1α* and cell death-inducing DFFA like effector A (*Cidea*), reported by about 15- (*p* < 0.01) and 77-fold (*p* < 0.001) significantly enhanced gene expression. No similar effects were found at thermoneutrality. We also observed higher expression of *Pgc1α* and *Cidea* in the SAT of EPA-fed WT and KO mice at both temperatures, but no significant differences were observed (Figure 4B,C), which explain the slight beneficial effect of EPA on beige adipocytes programming. To investigate the factors, which may associate with temperature-dependent DIO resistance, other than UCP1, we measured the gene expression of commonly known batokines, like fibroblast growth factor 21 (*Fgf21*) and bone morphogenetic protein 8b (*Bmp8b*), which were significantly upregulated by the absence of UCP1 by 16- and 15-fold only at ambient temperature but not at thermoneutrality (Figure 4D,E). Importantly, in UCP1 KO mice, EPA significantly increased the *Fgf21* gene expression (*p* = 0.0012) compared to the HFD at ambient temperature. A significant effect of temperature (*p* < 0.0001) and genotype (*p* < 0.005) and interaction between temperature and genotype were observed for the gene expression levels of *Ucp1*, *Pgc1α*, *Cidea*, *Fgf21*, and *Bmp8b* (Table 1).

**Figure 5:**
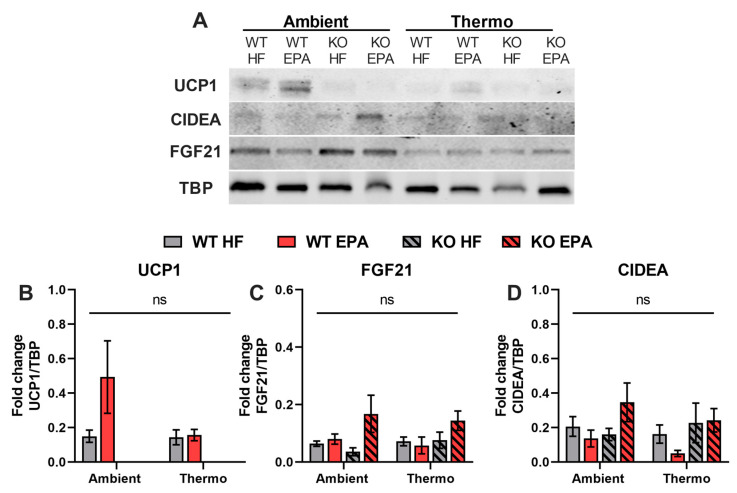
Effects of UCP1 and temperature on thermogenic protein expression levels in the SAT of WT and UCP1 KO mice fed an HFD or EPA-supplemented diet. (**A**) The protein content of UCP1, CIDEA, and FGF21 in the SAT of mice, (**B**–**D**) blots were normalized to TBP. Data are expressed as mean ± SEM; figures with no letters indicate no statistical (ns) differences analyzed by three-way ANOVA across the groups, n = 4–5.

We further evaluated protein expression levels of UCP1, CIDEA, and FGF21 in different groups (Figure 5A). As expected, consistent with gene expression, UCP1 was undetectable in KO groups. While in the WT mice, compared to the HFD group, EPA group showed a 3-fold increase in UCP1 content at ambient temperature and comparable UCP1 content in thermoneutrality (Figure 5B). As a key adipogenic transcription factor, the protein expression level of CIDEA was remarkably enhanced by EPA, compared to the HFD, in KO mice at ambient temperature (Figure 5C). We also measured the protein content of FGF21 and found that WT mice fed with EPA have comparable amounts of FGF21 to those found in HFD-fed mice at both temperatures. In KO mice, however, EPA upregulated FGF21 content by 4.7- and 2.1-fold at ambient and thermoneutral temperatures (Figure 5D), which further supported the UCP1-independent molecular pathway of beige cell programming in SAT.

### 2.5. Effects of EPA and UCP1 Deficiency on SAT Browning, Lipid Metabolism, and Alternative Thermogenesis Temperature-Dependently

**Figure 6:**
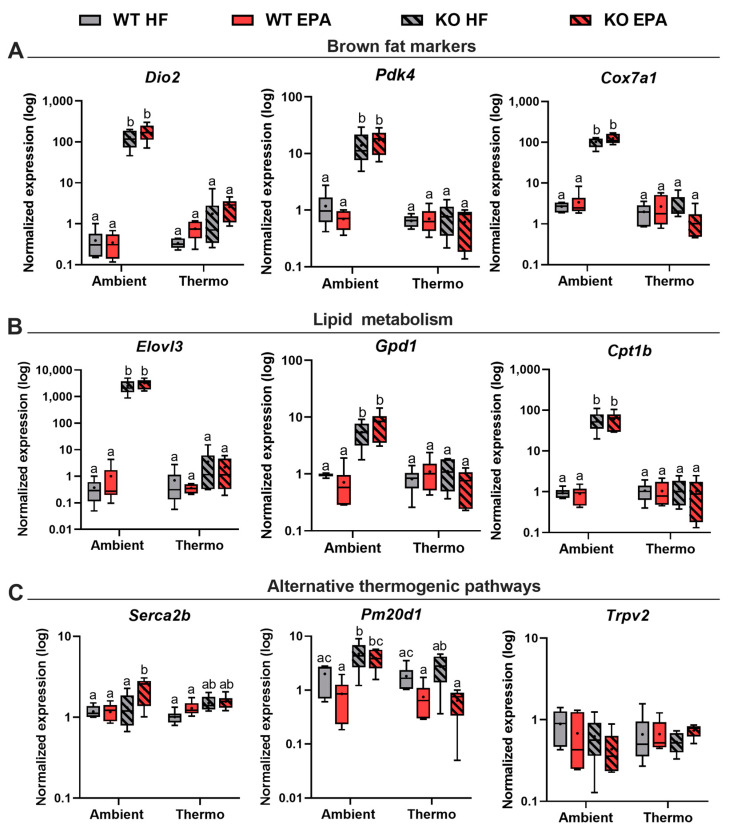
Effects of UCP1 and temperature on thermogenic, lipid metabolic, and alternative thermogenic gene expression levels in the SAT of WT and UCP1 KO mice fed an HFD or EPA-supplemented diet. The expression levels of genes involved in (**A**) brown/beige fat biogenesis, (**B**) lipid metabolism, and (**C**) alternative thermogenic pathway. Box plots show the distribution of log2 gene expression levels. Groups represented with different letters indicate significant difference reported by three-way-ANOVA, *p* < 0.05, n = 6.

Next, to validate the molecular signatures of SAT browning, the gene expression of well-identified batokines and brown fat markers were quantified. Upon ambient temperature, there were in the SAT of UCP1 KO mice 317-, 12-, and 40-fold significant increases in key thermogenic genes: iodothyronine deiodinase 2 (*Dio2*), pyruvate dehydrogenase kinase 4 (*Pdk4*), and cytochrome c oxidase 7a1 (*Cox7a1*) expression (*p* < 0.0001, *p* = 0.0002, *p* < 0.0001), respectively, compared to WT mice (Figure 6A). No changes in the gene expression of the above thermogenic markers between WT and UCP1 KO mice were observed at thermoneutrality. In response to EPA, UCP1 KO mice housed at ambient temperature expressed higher levels of *Dio2*, *Pdk4*, and *Cox7a1* than HFD-fed KO mice, although no significant differences were observed. Similar to browning-related genes, the SAT in KO mice maintained at ambient temperature expressed higher levels of genes sensitive to cold and genes involved in lipid metabolism (Figure 6B). As an important regulator for early onset of lipid recruitment [22], mRNA levels of the fatty acyl chain elongase (*Elovl3*) were elevated 1000-fold in KO mice (*p* < 0.0001) in comparison to WT mice at ambient temperature. Additionally, the gene expression of glycerol 3-phosphate dehydrogenase 1 (*Gpd1*) and carnitine palmitoyl transferase 1b (*Cpt1b*), involved in lipogenesis and fatty acid oxidation, were also 7- and 63-fold higher in the SAT from ambient-exposed KO mice (*p* < 0.001; *p* < 0.0001) than in WT mice, reflecting the induction of browning in SAT in UCP1 independent manner. However, neither diet nor the absence of UCP1 altered the lipid metabolism pattern of mice at thermoneutrality. Importantly, compared to the HFD, EPA upregulated the mRNA expression levels of the above genes in both WT and KO mice only at ambient temperature but not at thermoneutrality, revealing the temperature-dependent enrichment of lipid and oxidative metabolism with EPA supplementation. Finally, UCP1 KO mice featured obesity resistance at ambient temperature, suggesting the existence of alternative pathways of thermogenesis. Therefore, genes of recently identified UCP1-independent thermogenic pathways were quantified. At ambient temperature, the mRNA level of sarco/endoplasmic reticulum Ca^2+^-ATPase 2b (*Serca2b*) was significantly increased by EPA (*p* = 0.012) independently of UCP1, indicating the noncanonical thermogenic potential of EPA via enhancing ATP-dependent Ca^2+^ cycling pathway [14]. We also found that the expression level of peptidase M20 domain containing 1 (*Pm20d1*), which generates n-acyl amino acid as endogenous uncouplers [15], was significantly higher in the KO mice than WT mice fed both an HFD (*p* = 0.032) and an EPA (*p* = 0.009) diet only at ambient environment. The last gene involved in the alternative thermogenesis we quantified was transient receptor potential vanilloid 2 (*Trpv2*) [23]; however, no difference in expression was observed among all groups (Figure 6C). Taken together, our results demonstrate that the expression levels of genes relevant to beige adipocytes programming in the SAT were dramatically enhanced in response to the absence of UCP1 only at ambient temperature. Further, EPA has potential beneficial effects on SAT browning in response to temperature and UCP1 independently. A significant effect of temperature (*p* < 0.0001) and genotype (*p* < 0.0001) and the interaction between temperature and genotype (*p* < 0.0001) were observed for the gene expression levels of browning markers and lipid metabolism (Table 1). At last, we performed an analysis of the fatty acid profile in the SAT from all groups (Table S1). As expected, EPA was only found in the SAT of EPA-fed mice, and no significant differences were observed between genotypes and temperatures.

### 2.6. Effects of EPA and UCP1 Deficiency on Browning and Respiration Capacity in Cultured Primary Adipocytes

**Figure 7:**
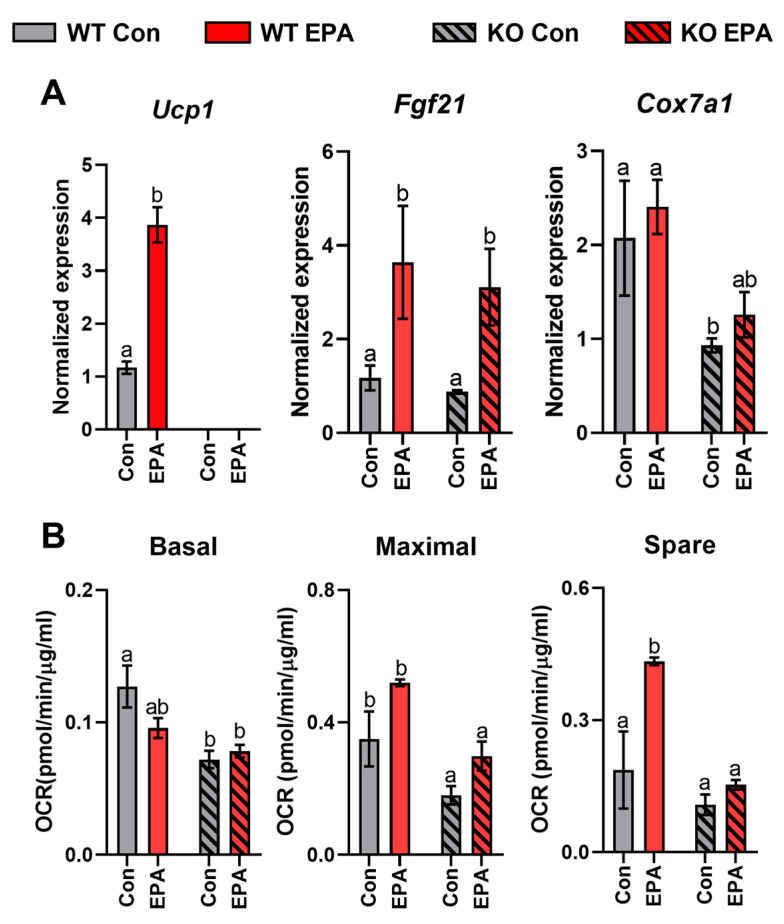
Effects of UCP1 and EPA on thermogenic genes expression and respiration capacity of SAT-derived adipocytes from WT and UCP1 KO mice. (**A**) The expression levels of thermogenic genes and (**B**) respiration capacity of differentiated adipocytes treated with vehicle control (Con) or EPA. Data were expressed as mean ± SEM and analyzed by two-way ANOVA with different letters indicating significant difference, *p* < 0.05, three independent studies.

To further validate the role of UCP1 in thermogenesis and determine how EPA regulates the beige adipocytes programming in absence of UCP1, we cultured differentiated SAT-derived primary adipocytes in WT and UCP KO male mice (Figure 7A). In response to EPA treatment, compared to the control, the mRNA level of *Ucp1* was significantly increased in the WT group (*p* = 0.0003), and as expected, *Ucp1* levels were undetectable in the KO group, which was consistent with the animal study. Additionally, classic batokines, such as *Fgf21*, and well-established browning markers, such as *Cox7a1*, were upregulated by EPA in both genotypes, which suggested that EPA can induce beige cell formation during adipocyte differentiation, UCP1-independently. Other genes involved in browning regulation (*Pgc1α*, *Prdm16*, *Pparγ*, and *Bmp8b*) and lipid metabolism (*Elovl3* and *Cpt1b*) were also increased by EPA in both genotypes, but no significant differences were observed (Figure S1, Table S2). Then, we performed mitochondrial function analysis by measuring the oxygen consumption rate (OCR) to investigate whether the above EPA-induced enhancement of thermogenic markers is responsible for the change of mitochondrial oxidative phosphorylation rate in differentiated adipocytes (Figure 7B). As expected, the absence of UCP1 decreased mitochondrial respiration, highlighting the important role of UCP1 in mitochondrial function. Basal respiration in WT and KO adipocytes was not elevated by EPA, indicating the minor effect of EPA in ATP-linked respiration. However, the maximal respiration ORCs in WT and KO adipocytes experienced 48.6% and 66.7% increases with EPA, resulting in the 138% (WT, *p* = 0.0034) and 41.6% (KO) increase in spare respiratory capacity by EPA. Additionally, the two-way ANOVA confirmed a significant main effect for treatment (*p* < 0.0001) and the interaction of treatment and genotype (*p* < 0.01) on maximal and spare respiration (Table 2). Taken together, EPA exerts appreciable effects on several parameters of mitochondrial function in primary adipocytes UCP1 independently, which may contribute by the acquisition of a browning phenotype.

## 3. Discussion

Therapeutic activation and recruitment of thermogenic fats to increase energy expenditure and combat obesity have not been fruitful, due to the incomplete understanding of how physiological factors are integrated during the changes in environment, such as temperature and nutrients. Although UCP1 has been identified as a key thermogenic regulator, recently, thermogenic mechanisms beyond UCP1 have been uncovered in both brown and beige adipocytes [24]. Mice deficient in the UCP1 are a well-suited animal model to investigate UCP1-independent mechanisms and study human obesity since adults with obesity express only minor amounts of UCP1. In this study, as humans spend the vast majority of their lives at thermoneutrality, we studied both WT and UCP1 KO mice in the same condition. Comparing them with cold-adapted mice housed at ambient temperature, we show the indispensable role of temperature in mediating the phenotype of DIO mice and thermogenic profile in SAT independently of UCP1. The current study opened a new window to adults that obesity induced by UCP1-dependent thermogenic fat inactivation and depletion can be treated by hypothermal experience. Much evidence emphasizes the importance of omega-3 PUFA and metabolites in activating thermogenic fats and UCP1 expression. It has been noted that omega-3 PUFA induces brown and beige adipocyte differentiation, via the activation of G protein-coupled receptor 120 [25,26]. A more recent study reported that cold and β3-adrenergic stimulation promotes the release of 12-hydroxyeicosapentaenoic acid (12-HEPE), an omega-3 PUFA metabolite in mouse BAT to regulate cold adaptation and glucose metabolism [27]. However, the metabolic and thermogenic outcomes of omega-3 PUFA, similar to EPA, on brown or beige fat function of UCP1 independently are still unknown. In the current study, we focused on investigating the role of temperature in regulating UCP1-independent molecular networks of thermogenesis and the function of EPA in mediating beige adipocytes development in SAT. We demonstrate a genotypic difference in response to EPA on the DIO and SAT browning of male mice kept at different temperatures.

It should be noted that environmental temperature leads to a drastic alteration in the importance of UCP1 for metabolic outcomes in animal studies, such as body weight, adiposity, energy expenditure, and others. To “humanize” the thermal physiology of the mouse and mimic the thermoneutrality that humans live at, the thermoneutral temperature has been applied in mouse experiments [28]. The absence of UCP1 in B6 mice kept at thermoneutrality makes them prone to DIO, due to the lowest levels of heat generation to maintain homeothermy [6,29]. At sub-thermoneutral temperature, UCP1-deficient mice are resistant to DIO due to the activation of thermogenic mechanisms alternative to UCP1, which seem to be less efficient energy-wise, meaning that more energy is expended to produce the same amount of heat that UCP1 would produce [17,21,30]. Our study reproduced this robust phenomenon on HFD-fed UCP1 KO mice housed at ambient and thermoneutral temperatures paradoxically. Additionally, the UCP1 KO mice housed at ambient temperature, compared to WT mice, consumed more food and displayed a significant decrease in body weight in both diet groups. Consistent with our findings, a recent study characterized the impact of housing temperature on energy homeostasis and food intake. They observed that energy expenditure of DIO mice decreases by 30% from 22 °C to 30 °C without changing in food intake, leading to the higher body weight and fat at 30 °C [31]. On the other hand, although our data reveal no significant impact of EPA on food consumption, body weight, and adiposity, glucose clearance was enhanced in both the WT and UCP1 KO mice at both temperatures for the EPA-fed diet. In addition, although insulin tolerance was not different between the diets, EPA-fed mice were more insulin-sensitive, indicated by the reduced HOMA-IR. In response to the temperatures, beige adipocyte development and the activity of beige adipocytes were associated with systemic glucose homeostasis and insulin sensitivity [32,33]. Studies in animals and humans have reported that the dysfunction of thermogenic fat negatively impacts insulin resistance and T2D [34,35]. In line with the above studies, we observed a reduction in glucose tolerance and fasting insulin level in HFD-fed UCP1 KO mice at thermoneutrality. However, at ambient temperature, HFD-fed UCP1 KO mice have a comparable rate of glucose clearance with WT, along with similar levels of basal blood glucose and insulin. Given the browning of SAT in UCP1 KO mice at ambient temperature, the above beneficial effects may be mediated by newly discovered glycolytic beige adipocytes in the SAT [35]. 

The effect of EPA on reducing HFD-induced insulin tolerance is in part associated with the anti-inflammatory effect of EPA and the level of cytokines in serum. In agreement with our previous study [36], mice fed with EPA displayed higher plasma levels of adiponectin and decreased levels of leptin and resistin. As a insulin-sensitizing and anti-inflammatory protein secreted by white fat [37], several studies support an association between circulating adiponectin and the risk of developing T2D [38,39]. It was surprising that KO mice at thermoneutrality expressed higher adiponectin with the increase of body weight than WT mice, indicating the expression of adiponectin in KO mice improved insulin tolerance independently of weight change. Additionally, it has been proposed that the anti-inflammatory properties of EPA improves leptin sensitivity and reduce resistin levels [40]. Although leptin regulates feeding behavior and leptin deficiency mice show hyperphagia [41], circulating leptin positively correlates with fat mass, and leptin resistance occurs [42]. Additionally, resistin has been shown to induce insulin resistance in mice [43] and directly counter the anti-inflammatory effects of adiponectin [44]. We report remarkable decreases in leptin and resistin levels in mice fed with EPA at both temperatures, despite limited body weight reduction, which confirms that the anti-inflammatory effects of EPA are independent of UCP1, adiposity, and environment temperature. On the other hand, compared to WT, KO mice have lower leptin and resistin levels only at ambient temperature, possibly due to lower fat mass, but not at thermoneutrality. Collectively, these data delineate that the supplementation of EPA entirely mediates the insulin tolerance and obesity-induced inflammation in UCP1 and in a temperature-independent manner.

Our study pinpoints temperature as a crucial mediator of DIO resistance via SAT browning of UCP1 independently, and EPA accelerates this process. On the transcriptional level, genes identified as browning signatures, like *Cidea*, *Pgc1α*, and *Dio2*, were exclusively upregulated in the SAT of UCP1 KO mice housed at ambient temperature, demonstrating that the beige cells can be formatted without UCP1. Additionally, the upregulation of other genes involved in lipid metabolism, such as *Cpt1b* and *Pdk4*, and the respiratory chain, such as *Cox7a1* and *Gpd1*, elucidates temperature-induced lipid and glucose turnover [45] and possibly futile energy cycling [46]. Importantly, a recent study found that PM20D1 [15], an endogenous uncoupler, plays an important role in mediating metabolic profiles. Mice in the absence of PM20D1 are significantly more glucose intolerant and insulin resistant than the WT control in response to an HFD [47]. Our study, for the first time, reported that the gene expression of *Pm20d1* was selectively upregulated in UCP1 KO mice in a temperature-dependent manner. However, genes of calcium cycling (*Serca2b*) and calcium influx (*Trpv2*), with recently proposed alternative thermogenic mechanisms [21,48], were not affected by the absence of UCP1 in the current study. In the *in vitro* study, cells harvested from UCP1 KO mice expressed lower thermogenic genes than the cell harvested from WT mice. We also found that the absence of UCP1 leads to decreased oxygen consumption in differentiated adipocytes, due to the deficiency of UCP1-derived respiration [49]. The above evidence further confirmed the indispensable role of cold stimulation to induce SAT thermogenesis and protect mice from DIO without UCP1. On the aspect of EPA, Bargut et al. reported that male B6 mice supplemented with EPA as 2% of total energy at 20 °C induced markers of browning and thermogenic factors in SAT [50]. Additionally, in a human SAT-derived adipocyte culture study, 20μM EPA promotes beige adipogenesis by improving the mitochondrial function and the expression of *Ucp1* and *Cptb1* [51]. The above results suggest that EPA improves mitochondria activity and oxidation and thermogenesis in SAT [52]. In line with above evidence, our current study demonstrated that thermogenic effects of EPA were displayed in both WT and KO mice in a temperature-dependent manner. At first, EPA enhanced UCP1 expression (gene and protein) in the SAT of WT mice housed at ambient temperature. Similarly, in the KO mice housed in the same condition, we observed the pronounced induction of beige fat markers (CIDEA and FGF21) on both gene and protein levels. Many studies demonstrated that EPA significantly increased CIDEA expression level in differentiated human primary white [53] and brown adipocytes [54] to promote thermogenesis. Additionally, as a key energy homeostasis regulator, one study showed that FGF21 can be increased in obese women following a hypocaloric diet supplemented with EPA [55]. These data point to a potential role of EPA in inducing beige fat formation in humans, especially in obese humans with a limited amount of active UCP1. Secondly, to estimate the effect of EPA for beige adipocyte induction and mitochondrial function, we performed genes expression and respiratory capacity analysis in differentiated SAT-derived adipocytes with or without UCP1. In line with other studies, we observed that EPA increased *Ucp1* gene level in WT adipocytes, along with the enhancement of core thermogenic transcription factors and mitochondrial biogenesis. Importantly, EPA also enhanced the gene expression level of *Fgf21* in a UCP1-independent manner. Our findings in mitochondrial respiration exhibited that EPA has no impact on basal OCR in both WT and KO adipocytes, until injecting the uncoupler FCCP to mimic energy demand. Thus, the maximal and spare respiratory capacities of mitochondria in both WT and KO adipocytes were increased after EPA treatment. Other studies showed that mitochondrial content and function of fat were reduced in both obese humans [56] and rodents [57], and the content of omega-3 PUFA incorporated into fat was positively correlated with mitochondrial biogenesis, adipose tissue function, and obesity. 

## 4. Materials and Methods

### 4.1. Animal Study

Tissues used in this manuscript were from previously reported animal studies [19,20]. Briefly, inbred homozygous UCP1 KO mice and their WT littermate, which have been previously described, were used to perform experiments. Male mice were maintained at ambient temperature before the experimental procedures. At 5–6 week of age, WT and UCP1 KO mice were signed into different groups randomly (10–12 mice/ group) by housing at ambient or thermoneutral environment and feeding either an HFD or an HFD supplemented with 36 g/kg diet EPA-enriched fish oil (Alaskomega, Coshocton, OH, USA, Table S3). Mice have free access to food and water and were individually housed with a 12 h light/dark cycle. Weekly body weight and food intake were recorded. A glucose/insulin tolerance test and body composition were conducted during the feeding period. After 14 weeks intervention, mice were euthanized utilizing the CO_2_ inhalation method following 5 h fasting. Blood, SAT, and other tissues were harvested and stored at −80 °C for further analyses. Animal experiments were approved by the Texas Tech University Institutional Animal Care and Use Committee.

### 4.2. Glucose and Insulin Tolerance Tests

GTT and ITT were preformed after 11 and 12 weeks of dietary intervention along with 5 h fasting. Blood glucose levels were measured at 0, 15, 30, 60, 90, and 120 min after glucose (2 g/kg body weight) or insulin (1 U/ kg body weight, Humulin, Indianapolis, IN, USA) injection. OneTouch Ultra GlucoseMeter (AlphaTrack, North Chicago, IL, USA) was used for blood glucose measurement. The trapezoidal method was used to calculate area under the curve.

### 4.3. Measurement of Serum Hormone and Glucose Levels

Fasting serum glucose levels were measured using a GlucoseMeter, and serum hormones levels (leptin, resistin, adiponectin, and insulin) were detected using the metabolic hormones Milliplex kit (EMD Millipore Corporation, Burlington, MA, USA) in a multiplexing system (Luminex xMAP, Luminex Corporation, Austin, TX, USA). HOMA-IR was calculated by the following formula:

HOMA-IR = [fasting glucose (mg/dL) × fasting insulin (ng/mL)] × (405)^−1^ (1)

### 4.4. Body Composition

Body fat mass of mice was determined using an EchoMRI™ whole body composition analyzer (EchoMRI LLC, Houston, TX, USA).

### 4.5. SAT Fatty Acid Composition

Direct fatty acid methyl ester synthesis and gas chromatography/mass spectrometry methods were utilized to identify SAT fatty acid concentrations and to validate EPA delivery to the SAT. 

### 4.6. Mouse Stromal Vascular Fraction (SVF) Isolation, Maintenance, and Differentiation 

To get a single cell suspension, SAT from mice were weighed, minced, and digested by collagenase D (Sigma, St. Louis, MO, USA) and then filtered through 70μm nylon mesh (Spectrum, Rancho Dominquez, CA, USA). After centrifuging at 800× *g* for 10 min, SVF cells were washed, counted, and plated into 6-well plates with Dulbecco’s modified Eagle medium (DMEM; Thermo Fisher, Pittsburg, PA, USA) with 10% fetal bovine serum (Atlas Biologicals, Fort Collins, CO, USA) and 1× penicillin-streptomycin antibiotics (Thermo Fisher Scientific, Waltham, MA, USA). After 2 h, unattached cells were washed and removed with 1× phosphate-buffered saline (Sigma-Aldrich, St. Louis, MO, USA). SVF cells were cultured in the above medium until cells reached 80–90% confluence. Growth medium was mixed with 0.5 mM methylisobutylxanthine, 1 µM dexamethasone, and 10 µg/mL insulin (Sigma-Aldrich, St. Louis, MO, USA), and growth media with 10 µg/mL insulin with or without 100 μM EPA were used to induct and differentiate SVF cells. 

### 4.7. Mitochondrial Respiration

Mouse primary SAT-derived SVF was isolated from WT and UCP1 KO mice and seeded into 0.2% gelatin (wt/vol) coated 24-well XF cell culture microplates (Seahorse Bioscience, Billerica, MA, USA). Differentiated SVF cells were treated with or without 100 μM EPA. Then, culture media were changed to the XF assay media (Seahorse Bioscience, Billerica, MA, USA) containing 2 mmol/L sodium pyruvate and 25 mmol/L glucose and placed in a 37 °C non-CO_2_ incubator for 1 h. To determine oxygen consumption rate (ORC) changes in the differentiated SVF cells, an XF Cell Mito Stress Test Kit (Agilent, Santa Clara, CA, USA) was used and oligomycin A (1 mmol/L), carbonyl cyanide-4-(trifluoromethoxy) phenylhydrazone (FCCP, 0.3 mM), and antimycin/rotenone (A/R, 1 mM each) were injected according to the manufacturer’s instructions. Respiration profiles were calculated by the following formula:

Basal respiration = basal measurements − A/R measurements(2)

Maximal respiration = FCCP measurements − A/R measurements(3)

Spare respiration = maximal respiration − basal respiration(4)

### 4.8. RNA Isolation and Quantitative Real-Time PCR

SAT was homogenized in QIAzol^®^ reagent with a 4mm stainless bead using TissueLyser (Qiagen, Valencia, CA, USA). Total RNA from SAT or cells was extracted using Quick-RNA™ Miniprep Kit (Zymo Research, Irvine, CA, USA), and cDNA was prepared using the Maxima H Minus First Strand cDNA Synthesis Kit (Thermo Scientific, Grand Island, NY, USA). Gene expression was performed by real-time PCR by QuantStudio™ (Thermo Fisher Scientific, Waltham, MA, USA), and data were normalized against the housekeeping gene 18S. The primers used were designed using Oligoarchitech ™ Online software, purchased from Sigma-Aldrich (St Louis, MO, USA) and listed in Table S4. 

### 4.9. Western Blot

Proteins in SAT were extracted by lysing in modified radioimmunoprecipitation buffer (Thermo Fisher, Waltham, MA, USA), as previously described [19]. An equal amount of protein was loaded and separated using electrophoresis gels (Bio-Rad, Hercules, CA, USA) and then transferred to polyvinylidene fluoride membranes. Blocker™ FL Blocking Buffer (Thermo Fisher, Waltham, MA, USA) was used to block the membrane along with primary antibodies for TBP (1:1000, Cell Signaling, Danvers, MA, USA) as a housekeeping control and UCP1 (1:1000, Thermo Fisher Scientific, Rockford, IL, USA), CIDEA (1:1000, Abcam, Cambridge, MA, USA) and FGF21 (1:1000, Abcam, Cambridge, MA, USA) as target genes. Rabbit polyclonal antibody was used as a secondary antibody (1:25,000). Blots were developed using Li-COR Imager System (Lincoln, NE, USA).

### 4.10. Statistical Analyses

Results were presented as means ± SEM). Data were analyzed by performing three-way ANOVA, including a main effect for temperature, diet, and genotype and their interactions. If significant, Tukey post hoc pairwise comparisons were conducted, and differences were considered significant at *p* < 0.05. All statistical tests were performed using GraphPad Prism software.

## 5. Conclusions

In summary, for the first time, we report detailed thermogenic effects of EPA in UCP1 deficient mice, which are temperature-dependent. Our findings suggest profound importance of temperature in inducing SAT browning. The paradoxical resistance to DIO in UCP1 KO mice appeared at ambient temperature and highlighted SAT browning as the driving force. Our analyses revealed the molecular induction of SAT browning of mice housed at ambient temperatures, suggesting compensatory thermoregulatory mechanisms, independent of UCP1. Surprisingly, EPA supplementation improved insulin resistance, systemic inflammation, and adipose thermogenesis regulation in both genotypes of mice during ambient acclimation. Our study is important to expand translational research on UCP1 alternative thermogenesis and dietary intervention to treat obesity and T2D. Further investigation of the molecular mechanisms that mediate UCP1-independent and EPA-mediated browning is warranted, including global transcriptomic and pathway analyses in the SAT in UCP1 KO mice in response to temperature and EPA. 

## Figures and Tables

**Table 1:** Effects of temperature (T), genotype (G), diet (D), and their interaction on metabolic characteristics and gene and protein expression in the SAT of mice reported by three-way ANOVA.

*p*-Value							
Variable	T (22 °C and 30 °C)	G (WT and KO)	D (HFD and EPA)	T×G	T×D	G×D	T×G×D
Weight gain (%)	**<0.0001**	**0.0148**	0.0865	**<0.0001**	0.6466	0.339	0.8248
Body fat (%)	**<0.0001**	0.3723	0.103	**0.0047**	0.7948	0.7401	0.3713
Food intake (g/week)	**<0.0001**	0.0919	0.2364	**0.0033**	0.3579	0.886	0.488
GTT (AUC)	0.5268	0.0562	**0.0057**	**0.0431**	0.5885	0.5501	0.6587
ITT (AUC)	0.8005	0.9539	**0.0379**	0.0802	0.2526	**0.0274**	0.1514
Basal glucose (mg/dL)	0.6747	0.1616	**0.0006**	0.5333	0.3693	**0.0007**	0.3628
Insulin (ng/mL)	0.1993	**0.0104**	**<0.0001**	**0.0252**	0.7561	0.7338	0.9571
HOMA-IR	0.4693	**0.0147**	**<0.0001**	**0.0404**	0.8162	0.9545	0.4613
Adiponectin (ng/mL)	**<0.0001**	**0.0005**	**<0.0001**	**0.0003**	0.3458	0.2601	0.3713
Leptin (ng/mL)	**<0.0001**	**<0.0001**	**<0.0001**	**<0.0001**	**0.0029**	**0.0001**	**0.0026**
Resistin (ng/mL)	**<0.0001**	**0.0353**	0.0532	0.029	0.3747	0.4758	0.6235
*Gene expression*							
*Ucp1*	0.7772	**0.004**	**0.0144**	0.7801	0.9736	**0.0143**	0.9706
*Pgc1α*	**0.0006**	**<0.0001**	0.1064	**<0.0001**	0.5053	0.1846	0.9336
*Cidea*	**<0.0001**	**<0.0001**	0.1263	**<0.0001**	0.1641	0.1435	0.1497
*Fgf21*	**<0.0001**	**<0.0001**	**0.0011**	**<0.0001**	**0.0031**	**0.002**	**0.0064**
*Bmp8b*	**<0.0001**	**<0.0001**	0.2358	**<0.0001**	0.2913	0.2525	0.2099
*Dio2*	**<0.0001**	**<0.0001**	0.1958	**<0.0001**	0.2177	0.2021	0.2093
*Pdk4*	**<0.0001**	**<0.0001**	0.5756	**<0.0001**	0.5449	0.4632	0.4111
*Cox7a1*	**<0.0001**	**<0.0001**	0.2321	**<0.0001**	0.1887	0.2998	0.189
*Elovl3*	**<0.0001**	**<0.0001**	0.3651	**<0.0001**	0.3628	0.3655	0.3641
*Gpd1*	**<0.0001**	**<0.0001**	0.3299	**<0.0001**	0.276	0.3456	0.1151
*Cpt1b*	**<0.0001**	**<0.0001**	0.8621	**<0.0001**	0.848	0.8579	0.8456
*Serca2b*	0.2977	**0.0004**	**0.0164**	0.3639	0.2681	0.1648	**0.0274**
*Pm20d1*	**0.0013**	**0.0002**	**0.0027**	**0.0036**	0.4476	0.6811	0.3878
*Trpv2*	0.9402	0.1674	0.6547	0.2659	0.1398	0.5885	0.6588
*Protein expression*							
UCP1	0.3755	0.0632	0.432	0.3755	0.4666	0.432	0.4666
FGF21	0.9769	0.2758	0.1465	0.8395	0.4888	0.1625	0.8169
CIDEA	0.9203	0.3463	0.99	0.7359	0.6353	0.4265	0.8709

WT, wild-type; KO, UCP1 knockout; HFD, high fat diet; EPA, HFD supplemented with EPA.

**Table 2:** Effects of genotype, treatment, and their interaction on gene expression and respiration in differentiated primary adipocytes reported by two-way ANOVA.

*p*-Value			
Variable	Genotype (WT and KO)	Treatment (Con and EPA)	Genotype × Treatment
*Gene expression*			
*Ucp1*	**<0.0001**	**0.001**	**0.001**
*Fgf21*	0.5473	**0.0026**	0.8653
*Cox7a1*	**0.0048**	0.3722	0.9931
*Respiration*			
Basal	**0.0026**	0.2499	0.0895
Maximal	**<0.0001**	**<0.0001**	**0.0059**
Spare	**<0.0001**	**<0.0001**	**<0.0001**

WT, wild-type; KO, UCP1 knockout; Con, vehicle control; EPA, 100μM EPA in cell culture medium.

## Data Availability

All of the data supporting this work will be made available from the corresponding author upon reasonable request.

## Supplementary Materials

The supporting information can be downloaded at: https://www.mdpi.com/article/10.3390/ijms24108708/s1.
