# Supplementary material for: Temperature-Dependent Effects of Eicosapentaenoic Acid (EPA) on Browning of Subcutaneous Adipose Tissue in UCP1 Knockout Male Mice
Source: Int J Mol Sci. 2023 May 13;24(10):8708. doi: 10.3390/ijms24108708 (PMC10218247; doi:10.3390/ijms24108708)
Supplement: Supplementary file 1 [file ijms-24-08708-s001.zip › ijms-2337183-supplementary.pdf]

Supplementary materials

Temperature-Dependent Effects of Eicosapentaenoic Acid (EPA) on Browning of Subcutaneous Adipose Tissue in UCP1 Knockout Male Mice

Table S1. Effects of temperature (T), genotype (G), diet (D) and interaction on fatty acid composition (%) of SAT in WT and KO mice reported by three-way ANOVA at ambient (A) and thermoneutral (T) temperatures; groups represented with different letter indicate significant difference reported by three-way ANOVA, n = 4.

	**Group**						**Three-way ANOVA**						
	**Temp**	**WT HF**	**WT EPA**	**KO HF**	**KO EPA**		**T** (22°C and 30°C)	**G** (WT and KO)	**D** (HFD and EPA)	**T×G**	**T×D**	**G×D**	**T×G×D**
**Palmitic acid**	**A**	17.16	18.72	18.06	18.28	**p**	**<0.0001**	**0.0179**	**0.001**	0.1336	0.9131	0.1706	0.154
16:0	**T**	19.19	20.02	20.12	20.98	**F**	75.87	6.466	14.01	2.411	0.01217	1.996	2.167
**Palmitoleic acid**	**A**	1.08	0.93	1.19	1.16	**p**	**0.0017**	**0.001**	**0.0088**	0.2274	0.8527	**0.0499**	0.8237
16:1 (n-7)	**T**	1.02	0.84	1.03	1.00	**F**	12.45	14.09	8.141	1.535	0.03524	4.264	0.05074
**Linoleic acid** 18:2	**A**	21.59	20.97	19.65	19.71	**p**	0.7638	**<0.0001**	0.3283	0.5309	0.0613	0.9677	0.2799
(n-6)	**T**	20.42	21.59	19.50	20.05	**F**	0.09234	23.83	0.9957	0.4042	3.855	0.001677	1.222
**α-linolenic acid**	**A**	0.99	1.07	0.86	0.85	**p**	**<0.0001**	0.7276	0.0529	**<0.0001**	0.3188	0.8768	0.1956
18:3 (n-3)	**T**	1.15	1.21	1.27	1.40	**F**	96.6	0.1242	4.146	27.12	1.037	0.02453	1.773
**EPA**	**A**	**0.00** ^{a}	**2.97** ^{b}	**0.00** ^{a}	**2.04** ^{b}	**p**	**0.0153**	0.3089	**<0.0001**	**0.0002**	**0.0153**	0.3089	**0.0002**
20:5 (n-3)	**T**	**0.00** ^{a}	**2.49** ^{b}	**0.00** ^{a}	**4.00** ^{b}	**F**	6.825	1.081	417.7	18.88	6.825	1.081	18.88
**DHA**	**A**	**0.10** ^{a}	**0.66** ^{b}	**0.13** ^{a}	**0.70** ^{b}	**p**	0.2891	**0.0354**	**<0.0001**	0.1954	0.8782	0.3315	0.4112
22:5 (n-3)	**T**	**0.12** ^{a}	**0.62** ^{b}	**0.19** ^{a}	**0.84** ^{b}	**F**	1.175	4.973	196	1.774	0.02398	0.9824	0.6994

WT, wild-type; KO, UCP1 knockout; HFD, high fat diet; EPA, HFD supplemented with EPA.

We performed an analysis of fatty acid profile in subcutaneous adipose tissue (SAT) from all groups (Table S1). EPA was only found in the SAT of EPA fed mice (WT and KO) at both temperatures, and the three-way ANOVA revealed a significant effect for diet ($p <$ 0.0001) and interaction between diet, temperature, and genotype ($p = 0.002$). Compared to the HFD, EPA increased the percentage of DHA in WT and KO mice by 6.5 and 5.3-folds at ambient temperature and by 5.0 and 4.9-folds at thermoneutrality, respectively. In addition, the percentage of DHA in UCP1 KO mice was slightly higher than it in WT mice at both temperatures. The three-way ANOVA revealed a significant main effect for diet ($p <$ 0.0001) and genotype ($p = 0.0354$) on DHA percentage in SAT. Both WT and KO mice at thermoneutrality have significantly higher percentage of α-linolenic acid, another omega-3 fatty acid, in SAT compared to mice at ambient temperature. In addition, the three-way ANOVA revealed a significant main effect on temperature, genotype, or diet for both palmitic and palmitoleic acid. Compared to the ambient temperature, both WT and KO mice in thermoneutrality have significantly higher percentage of palmitic acid, but lower percentage of palmitoleic acid, in the SAT. Interestingly, compared to the HF, both WT and KO mice fed with EPA have higher percentage of palmitic acid, but lower percentage of palmitoleic acid, in the SAT at both temperatures. No significant differences were observed in the percentages of linoleic acid among the groups.

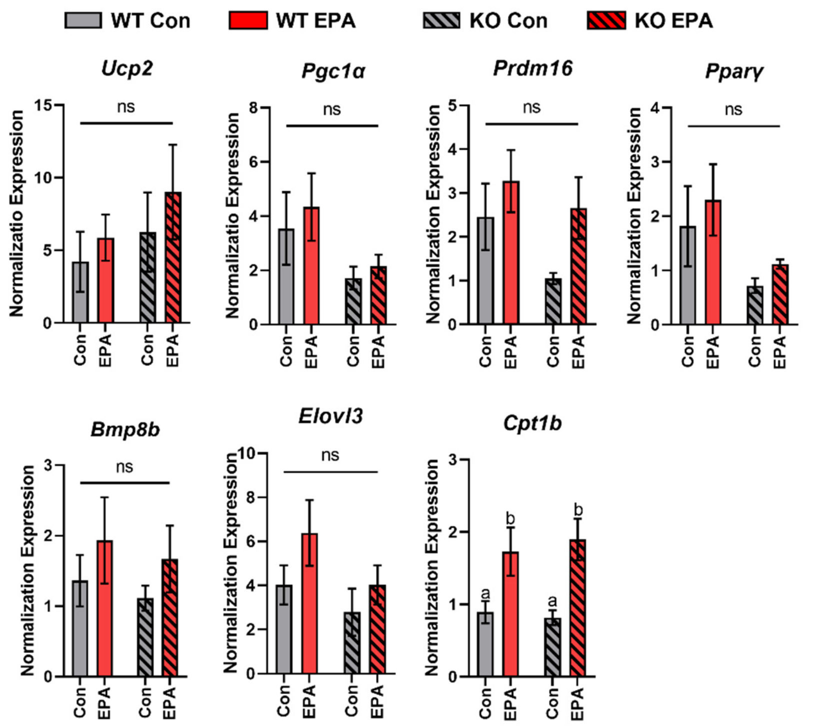

Figure S1. mRNA expression of thermogenic genes regulated by EPA in primary subcutaneous adipocytes isolated from WT and UCP1 KO mice. Data were expressed as mean ± SEM and analyzed by two-way ANOVA with different letters indicating significant difference, $p < 0.05$, three independent studies.

Table S2. Effects of genotype, treatment and interaction of G and T on gene expression and respiration in differentiated primary adipocytes reported by two-way ANOVA.

	p-Value		
**Variable**	**Genotype** (WT and KO)	**Treatment** (Con and EPA)	**Genotype × Treatment**
Ucp2	0.3188	0.3935	0.8287
Pgc1α	**0.0497**	0.5342	0.8506
Prdm16	0.1396	0.0782	0.577
Pparγ	**0.0342**	0.3887	0.9305
Bmp8b	0.5654	0.2109	0.9865
Elovl3	0.1211	0.1211	0.622
Cpt1b	0.8491	**0.0007**	0.6171

WT, wild-type; KO, UCP1 knockout; Con, saline control; EPA, 100 μM EPA in cell culture medium.

We differentiated SAT-derived primary adipocytes isolated from WT and UCP KO male mice to validate the role of UCP1 in thermogenesis and determine how EPA regulates the beige adipocytes programming in absence of UCP1. Genes involved in thermogenesis (*Ucp2*), browning regulation (*Pgc1α*, *Prdm16*, *Pparγ* and *Bmp8b*) and lipid metabolism (*Elovl3* and *Cpt1b*) were increased by EPA in both genotypes (Figure S1), but no significant difference were observed (Table S2).

Table S3. Diet composition.

			D08112003	
	**HF-EPA**		**HF**	
	gm	kcal (%)	gm	kcal (%)
Protein	24	20	24	20
Carbohydrate	41	35	41	35
Fat	24	45	24	45
Total		100		100
	gm	kcal	gm	kcal
Casein	200	800	200	800
L-Cystine	3	12	3	12
Corn Starch	72.8	291	72.8	291
Maltodextrin 10	100	400	100	400
Sucrose	172.8	691	172.8	691
Cellulose	50	0	50	0
Soybean Oil	25	225	25	225
Lard	139.5	1256	177.5	1598
AlaskOmega EE (820 mg EPA/g)	38	32	0	0
Mineral Mix S10026	10	0	10	0
Di Calcium Phosphate	13	0	13	0
Calcium Carbonate	5.5	0	5.5	0
Potassium Citrate, 1 H2O	16.5	0	16.5	0
Vitamin Mix V10001	10	40	10	40
Choline Bitartrate	2	0	2	0
Vitmain E Acetate, 50% (500IU/gm)	0.13	0	0.13	0
FD&C Yellow Dye #5	0.05	0	0	0
FD&C Red Dye #40	0	0	0.025	0
FD&C Blue Dye #1	0	0	0.025	0
Total	858.27	4057	858.28	4057
EPA content in the diet (g/kg diet)	36		0	

Table S4. Primer sequences.

Gene	Forward (5´-3´)	Reverse (5´-3´)
Ucp1	GAGGTGTGGCAGTGTTCATT	CTGTGGTGGCTATAACTCTGTAAG
Pgc1α	GAGCGAGTGAGCGTGAGA	GTGGCAGTGGAACATCAACA
Cidea	TCCTCGGCTGTCTCAATGT	GATGGCTGCTCTTCTGTATCG
Bmp8b	GCTCAGGCTCTTCAGTATC	ACAGGCTATGGAGTGCTA
Dio2	TGCGCTGTGTCTGGAACAG	CTGGAATTGGGAGCATCTTCA
Pdk4	TGTGATGTGGTAGCAGTAGT	TGGTGAAGGTGTGAAGGA
Cox7a1	GTGGCAGAGAAGCAGAAG	CCAAGCAGTATAAGCAGTAGG
Elovl3	ACGGAACACTTCTTCTGGTCTT	CCTACTCTCATTGGCTCTTGGA
Gpd1	CGGAGACAAGCGGAAGGA	CCAGAGGACAGCAAGGAAGT
Cpt1b	CCACAGACAGAGGCACTT	CAGGAGACGGACACAGATAG
Serca2b	ACCTTTGCCGCTCATTTTCCAG	AGGCTGCACACACTCTTTACC
Pm20d1	AAAGAGGCGCTGAAAGGTG	GTGGCTGAAAGACACTGTGG
Trpv2	TTGACCGTGACCGACTCTT	GGCAGGATACAGGCATTGAC
